# Genomic structural variations for cardiovascular and metabolic comorbidity

**DOI:** 10.1038/srep41268

**Published:** 2017-01-25

**Authors:** Maria S. Nazarenko, Aleksei A. Sleptcov, Igor N. Lebedev, Nikolay A. Skryabin, Anton V. Markov, Maria V. Golubenko, Iuliia A. Koroleva, Anton N. Kazancev, Olga L. Barbarash, Valery P. Puzyrev

**Affiliations:** 1 Laboratory of Population Genetics, Research Institute of Medical Genetics, Tomsk National Research Medical Center, Russian Academy of Sciences, Tomsk, Russia; 2 Laboratory of Human Ontogenetics, Tomsk State University, Tomsk, Russia; 3 Laboratory of Cytogenetics, Research Institute of Medical Genetics, Tomsk National Research Medical Center, Russian Academy of Sciences, Tomsk, Russia; 4 Department of Experimental and Clinical Cardiology, Research Institute for Complex Issues of Cardiovascular Diseases, Kemerovo, Russia; 5 Department of Multifocal Atherosclerosis, Research Institute for Complex Issues of Cardiovascular Diseases, Kemerovo, Russia

## Abstract

The objective of this study was to identify genes targeted by both copy number and copy-neutral changes in the right coronary arteries in the area of advanced atherosclerotic plaques and intact internal mammary arteries derived from the same individuals with comorbid coronary artery disease and metabolic syndrome. The artery samples from 10 patients were screened for genomic imbalances using array comparative genomic hybridization. Ninety high-confidence, identical copy number variations (CNVs) were detected. We also identified eight copy-neutral changes (cn-LOHs) > 1.5 Mb in paired arterial samples in 4 of 10 individuals. The frequencies of the two gains located in the 10q24.31 (*ERLIN1*) and 12q24.11 (*UNG, ACACB*) genomic regions were evaluated in 33 paired arteries and blood samples. Two patients contained the gain in 10q24.31 (*ERLIN1*) and one patient contained the gain in 12q24.11 (*UNG, ACACB*) that affected only the blood DNA. An additional two patients harboured these CNVs in both the arteries and blood. In conclusion, we discovered and confirmed a gain of the 10q24.31 (*ERLIN1*) and 12q24.11 (*UNG, ACACB*) genomic regions in patients with coronary artery disease and metabolic comorbidity. Analysis of DNA extracted from blood indicated a possible somatic origin for these CNVs.

Cardiovascular diseases and metabolic syndrome are closely interrelated via pathogenesis and considered complex comorbid disorders. Metabolic abnormalities, such as abdominal obesity, high blood pressure, hyperglycaemia, low high-density lipoprotein cholesterol, and elevated triglycerides, are important risk factors for cardiovascular diseases. Metabolic syndrome is associated with advanced vascular damage and complicated atherosclerotic lesions in patients with coronary artery disease^1^.

The role of genetic variability in the pathogenesis of both coronary artery disease and metabolic syndrome has been extensively investigated during recent decades. Genetic polymorphisms identified through candidate gene or genome-wide association studies account for a small part of the heritability of these complex traits. Possible factors that may affect some of the “missing heritability” include structural genomic variants, rare variants, epigenetic mechanisms, gene-gene interactions, and gene-environment interactions^2^.

The advent of genome-wide high throughput technologies has allowed the characterization of structural genomic variants, such as copy number variants (CNVs), in a single array experiment. Several CNVs explain rare inherited disorders, and other CNV events are thought to be involved in the aetiology and pathogenesis of complex traits and diseases. There is also emerging evidence that somatic genetic changes are more common than previously predicted^3^,^4^. For example, somatic gene alterations have been observed in multiple tissues^5^,^6^,^7^. The study of somatic DNA copy number and copy-neutral changes is important for cancer genomics; however, to date, the evidence for their involvement in other diseases remains limited.

The studies of CNVs in coronary artery disease have typically used DNA isolated from peripheral blood, and inconclusive results were obtained^8^,^9^,^10^,^11^. The most pronounced genomic imbalance in patients with coronary artery disease and metabolic comorbidity are possibly detected in the target disease organs, such as coronary arteries. Plaques potentially develop via a series of somatic mutations, and atherosclerosis has similarities to malignant diseases^12^. Chromosomal aberrations, loss of heterozygosity (LOH), microsatellite instability (MI), and DNA strand breaks and adducts were detected within human atherosclerotic plaques^13^,^14^. However, a comprehensive profile of the DNA differences between atherosusceptible and atheroprotected arterial regions has yet to be developed. Most of these studies have used traditional cytogenetic methods, which have low resolution and require cells that are actively dividing^15^. Additionally, only selective candidate microsatellite DNA loci were tested for the detection of LOH and MI in the area of atherosclerotic plaques^16^,^17^,^18^,^19^,^20^,^21^,^22^,^23^.

We sought to identify the genes targeted by the both copy number and copy-neutral changes in the right coronary arteries in the area of advanced atherosclerotic plaques (CAP) and in intact internal mammary arteries (IMA) derived from the same individuals with coronary artery disease and metabolic syndrome. The study of coronary artery-specific CNV regions of patients with coronary artery disease and metabolic comorbidity may improve our knowledge of comorbidity disease mechanisms.

## Results

In our analysis of 10 individuals using the Agilent SurePrint G3 Human CGH + SNP Microarray 2 × 400 K assay, we identified 90 CNVs present in both tissue samples obtained from a single individual (Table S1). The average number of CNVs per individual was 38.4, ranging from 31 to 46. The average CNV size was approximately 217 kb, ranging from 4.4 kb to 2 Mb. Most of the observed CNVs were copy number losses (62%). Fifty-five of 90 (61%) CNVs were present in more than one patient. Of the CNVs identified, 80% overlapped with RefSeq genes. The identified genes are associated with olfactory transduction and metabolic pathways, such as starch and sucrose metabolism, metabolism of xenobiotics by cytochrome P450, ascorbate and aldarate metabolism, and pancreatic secretion (Table S2).

**Figure 1:**
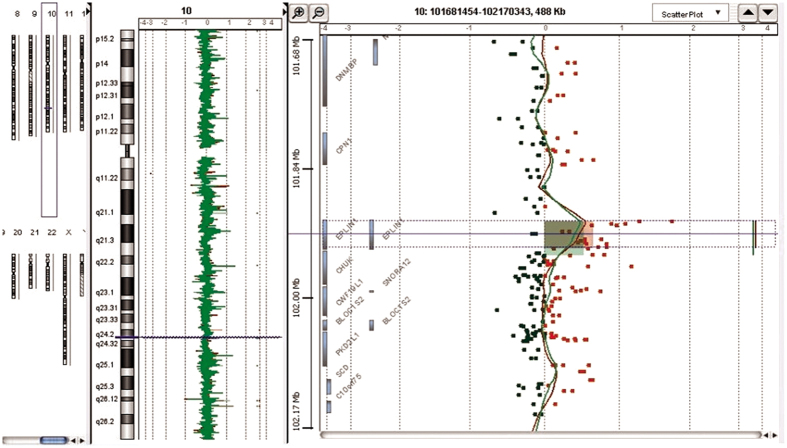
Array comparative genomic hybridization data analysis of patient 10. DNA of the right coronary artery in the area of advanced atherosclerotic plaque (red line) and DNA of the intact internal mammary artery (green line).

Fifteen chromosomal regions contained CNVs that are candidates for genes previously associated with cardiovascular and metabolic disease susceptibility (Table 1). One of the 15 CNVs was not listed in the DGV (accessed on 20.11.2015) and was therefore considered a novel CNV. One patient exhibited amplification in chromosomal region 10q24.31 (*ERLIN1*; Fig. 1).

The analysis of array data by both the Agilent Genomic Workbench and DNAcopy programs indicated no high-confidence coronary artery-specific copy number and copy-neutral changes when the DNA from CAP was compared with IMA.

Using the Agilent SurePrint G3 Human CGH + SNP Microarray 2 × 400 K, we also identified 8 copy-neutral changes (cn-LOH) present in both CAP and IMA samples (Table 2). These regions were detected in 4 of 10 individuals, spanning >1.5 Mb, with three changes located in chromosomal regions 7q21.12-q21.13, 11p12, 18q12.1-q12.2 (patient 1), 6q15, 8p11.21-p11.1, 8q11.1-q11.21 (patient 2), and one in 17q23.1-q24.1 (patient 3) and 9p21.2 (patient 7). Using the web-based GEne SeT AnaLysis Toolkit, we determined whether the genes located in cn-LOH are enriched for certain KEGG categories. The results indicated that genes fell into six KEGG categories: RIG-I-like receptor signalling pathway, neuroactive ligand-receptor interaction, Jak-STAT signalling pathway, Wnt signalling pathway, cell cycle, and ABC transporters (Table S3).

To validate the initial array-CGH data, we selected two genomic regions, 10q24.31 (*ERLIN1*) and 12q24.11 (*UNG, ACACB*), for qPCR analysis. The qPCR assay results were consistent with the array data.

**Figure 2:**
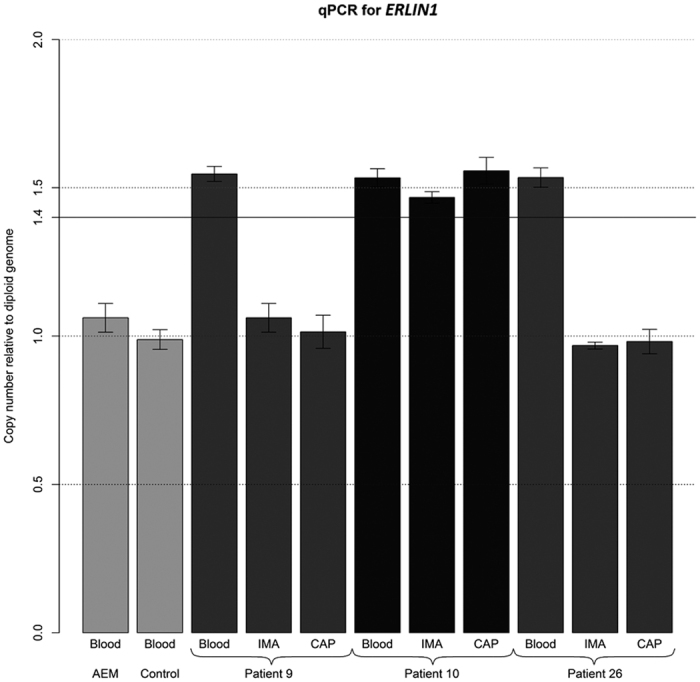
Real-time qPCR result of the CNV region on 10q24.31 in *ERLIN1*. Abbreviations: AEM - Human Reference DNA-Male with Caucasian ethnicity (5190-3796, Agilent Technologies, Santa Clara, CA, USA); CAP - right coronary artery in the area of advanced atherosclerotic plaque; IMA - intact internal mammary artery; qPCR - quantitative PCR.

**Figure 3:**
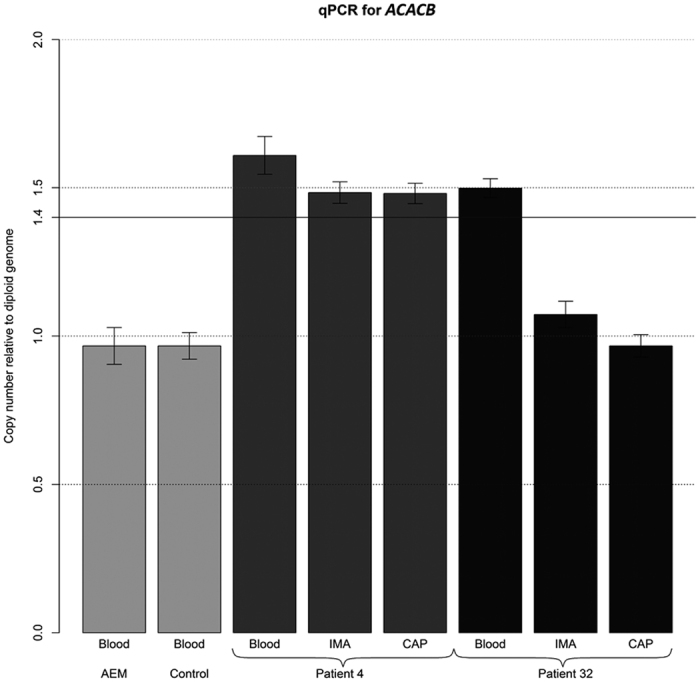
Real-time qPCR result of the CNV region on 12q24.11 in *UNG* and *ACACB*. Abbreviations: AEM - Human Reference DNA-Male with Caucasian ethnicity (5190-3796, Agilent Technologies, Santa Clara, CA, USA); CAP - right coronary artery in the area of advanced atherosclerotic plaque; IMA - intact internal mammary artery; qPCR - quantitative PCR.

To further validate our results, we evaluated the frequency of these CNVs in the DNA of 33 paired arteries and the blood of men with coronary artery disease and metabolic comorbidity. One patient exhibited increased gene copy numbers in 10q24.31 (*ERLIN1*) in all of his studied tissues (Fig. 2). Amplification of the same chromosomal region was also observed in the blood-derived DNA but was not present in the arteries-derived DNA (CAP and IMA) of two persons. Another patient contained the gain in region 12q24.11 (*UNG, ACACB*) in both the arteries (CAP and IMA) and blood (Fig. 3). One patient contained the gain in 12q24.11 (*UNG, ACACB*) that affected only the blood DNA.

## Discussion

Comprehensive knowledge of the genomic alteration events in cardiovascular diseases is critical for improved diagnostics and for developing targeted therapeutics. CNVs are a significant source of genetic diversity, but their influence on disease susceptibility remains poorly understood. For cardiovascular disease, several studies on CNVs in myocardial infarction and hyperlipidaemia patients have been reported^10^; however, there has been no CNV research for coronary artery disease and metabolic comorbidity. In the present study, we compared the genomic DNA in the CAP and IMA from patients with coronary artery disease and metabolic comorbidity using array-CGH. We used the Agilent SurePrint G3 Human CGH + SNP Microarray 2 × 400 K that allows for the simultaneous detection of copy number and copy-neutral changes on the same array across the entire genome.

Examination of the DNA in the arterial samples of 10 patients indicated the presence of 15 loci with CNVs that are candidates for genes previously associated with cardiovascular and metabolic disease susceptibility (Table 1). Most of these CNVs are reported recurrently in the DGV and are also present in the array-CGH database at the Laboratory of Cytogenetics, Institute of Medical Genetics. Therefore, these CNVs have a high likelihood of being benign and may represent polymorphisms.

We observed no candidate genes overlap between CNVs identified in our study and SNPs associated with coronary artery disease on a genome-wide level^24^. This result confirms the idea that CNVs could be new genetic markers to uncover susceptibility loci for future disease association studies.

In our study three different patients contained CNVs in genetic regions previously associated with coronary artery calcification in African Americans and carotid intima media thickness over 10 years in Chinese (2q37.3(*LOC105373979, LOC728323*), 1p22.2 (*GBP3*) and 10q21.1 (*PCDH15*))^25^,^26^.

The *GBP3* and *PCDH15* genes are a promising candidate for further study. Guanylate binding protein 3 (GBP3) belong to a family of proteins termed the guanylate-binding proteins (GBPs) that are induced in response to interferons. The function of these proteins has been primarily associated to protection against viral and bacterial infections^27^. GBPs may be also involved in the macrophage activation process with a potential role in atherogenesis. There was significant induction of *GBP3* gene expression in foam cells of advanced lesions of aortic sinuses isolated from *apoE*−/− mice fed Western diet for 14 weeks^28^,^29^.

Protocadherin 15 (*PCDH15*) encodes an integral membrane protein that mediates calcium-dependent cell-cell adhesion. So far nothing is known about the function of this protein in the arterial structure and function. *PCDH15* gene can be involved in the susceptibility to coronary artery disease via alteration of lipid metabolism. SNPs in *PCDH15* have been associated with triglycerides, apolipoprotein B and total cholesterol levels in familial combined hyperlipidemia^30^. In two patients CNV located 16q22.1 harboured amplification encompassing gene *PDPR*.

There is a hypothesis that amplification of *PDPR* gene might affect predisposition to coronary artery disease, while a loss of copy number may cause weaker recovery from heart attack due to alteration of protein function in the heart^31^.

One interesting finding is that the majority of CNVs located genes were associated with coronary artery disease risk factors but not with coronary artery disease directly (Table 1). For example, we found genetic losses at 1q31.3 (*CFHR3, CFHR1*), 3p21.1 (*SFMBT1*) and 22q11.23 (*GSTT1*). The SNPs of these genes were associated with blood pressure or hypertension in different populations^32^,^33^,^34^. Common deletion variant spanning *CFHR1* and *CFHR3* genes has been associated with complement over-activation, and increased inflammatory process^35^,^36^. *SFMBT1* encoding the Scm-like with four mbt domains protein 1 is involved in epigenetic regulation of gene expression via the histone methylation mechanism^37^. The molecular mechanism by which genetic variants of *SFMBT1* gene contribute to hypertension risk remains unknown. Сomplete deletion of the *GSTT1* gene lead to the complete absence of glutathione-S-transferase enzymatic activity and predispose to oxidative stress which implicated in the pathogenesis of hypertension^34^.

Obesity, type 2 diabetes mellitus and metabolic syndrome are important contributing causes of coronary artery disease. In our study one patient had the loss in region 9p21.1 (*LINGO2*). The leucine-rich repeat and Ig domain–containing 2 gene is expressed in neuronal tissue, and rs10968576 have been associated with body mass index^38^,^39^. This study also revealed CNV at 1p21.1 affecting *AMY2B* gene known to be associated with type 2 diabetes mellitus^40^. The pancreatic alpha-amylase gene codes for a protein that catalyzes the initial step in digestion of dietary starch and glycogen. CNVs in the *AMY2B* gene were significantly correlated with serum amylase levels and high amylase activity is related to improved glycemic homeostasis and lower frequencies of metabolic syndrome^41^. Finally, two of the deletions observed in the current study also fell into regions located at 7q33 and 16q13.12-q13.11, which is reported to harbour the metabolic syndrome candidate genes *EXOC4* and *PDXDC1*, respectively^42^,^43^.

To confirm the array CGH data, quantification of the dosage of genes present in amplifications at chromosomes 10q24.31 (*ERLIN1*) and 12q24.11 (*UNG, ACACB*) was performed by qPCR. These genes were selected for their potential functional roles in cardiovascular disease and risk factors associated with obesity, diabetes, and metabolic syndrome.

Amplification in chromosomal region 10q24.31 (*ERLIN1*) was novel compared with the DGV. ER lipid raft associated 1 (ERLIN1) is a component of the lipid rafts of the endoplasmic reticulum (ER)^44^. Lipid rafts are regions of the plasma membrane that act as platforms to colocalize proteins involved in intracellular signalling pathways. ERLIN1 is a cholesterol-binding protein that is directly involved in regulating the SREBP machinery^45^. ERLIN1 interacts with ERLIN2 to form a functional complex. A recent study of copy number variation in human breast cancer specimens demonstrated that *ERLIN2* is amplified and overexpressed in aggressive human breast cancer^46^. Increased expression of *ERLIN2* promotes the activation of the key lipogenic regulator SREBP1c and the production of cytosolic lipid droplets in breast cancer cells. The ERLIN1/ERLIN2 complex promotes ER-associated degradation of the activated IP3 receptor and 3-hydroxy-3-methylglutaryl-CoA reductase (HMGR), which is the well-known target of the statins^45^. SNPs near *ERLIN1* have previously been associated with the plasma levels of alanine aminotransferase and intracerebral haemorrhage in humans^47^,^48^. The mechanism of the increased copy number of *ERLIN1* in regulating cellular cholesterol homeostasis in patients with coronary artery and metabolic diseases must be further elucidated in future studies.

The gain CNV region in 12q24.11 occurs in *ACACB*, which encodes an enzyme with a wide range of important functions. The acetyl‐CoA carboxylase β enzyme is bound to the mitochondrial outer membrane. This enzyme produces malonyl-CoA that can be used either as a substrate for lipogenesis or as a negative modulator of mitochondrial fatty acid oxidation through its inhibition of carnitine palmitoyltransferase 1a. ACACB enzyme activity is controlled at the transcriptional level by several transcription factors, including peroxisome proliferator-activated receptors (PPARs) and sterol regulatory element-binding proteins (SREBPs), and is predominantly expressed in the heart and skeletal muscle. Mice deficient for *Acacb* exhibit increased overall and tissue-specific fatty acid oxidation^49^. Although originally shown to protect against diet-induced obesity and diabetes, follow-up studies observed that deletion of this gene upregulated mitochondrial fatty acid oxidation without altering overall energy homeostasis^50^. Cardiac-specific deletion of *ACACB* is sufficient to maintain fatty acid oxidation and prevent the metabolic remodelling that occurs during the development of pressure-overload hypertrophy^51^. At the juncture of lipid synthesis and oxidation pathways, inhibition of this enzyme presents a therapeutic target for metabolic syndrome. *ACACB* polymorphisms are associated with obesity, diabetes and metabolic syndrome in humans^52^. The gain in 12q24.11 (*ACACB*) was described in the DGV. The presence of this *ACACB* gain among the general population may indicate incomplete penetrance of the CNV, variable expression, or the presence of other genetic or environmental factors. Furthermore, many of the DGV individuals were recruited for different research studies not related to cardiovascular and metabolic disorders. It is possible that some of the gain carriers have ACACB overexpression and are affected by these disorders.

Somatic *de novo* genetic aberrations usually presented in the mosaic state are a common phenomenon in different tissues of healthy humans^7^. To the best of our knowledge, none of the previous studies compared the genomic alterations in atherosclerotic and intact arteries with those in the DNA extracted from the blood of the same individual. In the present study, we observed that two patients contained the gain in 10q24.31 (*ERLIN1*) and one patient contained the gain in 12q24.11 (*UNG, ACACB*) that affected only the blood DNA. Because the number of samples with a normal gene copy number exceeded the number of samples with gains, we hypothesize that an additional copy of a CNV region was added to a subset of leukocytes due postzygotic events, thereby resulting in somatic mosaicism. Notably, in this study, we measured the specific genomic alterations in a mixture of different cell types. Therefore, the fraction of cells in the arteries (e.g., macrophages) may also contain an undetected gain, such as low-level mosaicism. Further experiments in a larger cohort of patients and healthy persons using laser microdissection techniques and digital PCR are necessary to confirm our findings.

In addition to CNVs, we also detected areas of another type of genomic structural variations, such as cn-LOH. A wide range of cn-LOH regions was observed in leukocytes and different tissues of both healthy individuals and patients with cancer^53^,^54^. In our study, there were eight interstitial cn-LOH regions (>1.5 Mb) present in all studied arteries of four men with coronary artery disease and metabolic comorbidity. We suggest that these cn-LOH regions represent normal variations because they existed in all studied tissues of a patient. Six genes (*ABCB1, PLAT, ACE, GH1, PECAM1*, and *RGS9*) known to predispose people to coronary artery disease occur in the cn-LOH regions identified.

The results of this study demonstrate no significant evidence for high-confidence coronary artery-specific CNVs and cn-LOHs when the DNA from CAP was compared with IMA. It is possible that some existing somatic genomic structural variations may be missed in our study because of the limitations of the Agilent microarray platform with a gene-centric probe set and the strict analysis procedure used. Because we studied only 20 paired arterial samples derived from 10 patients, we cannot entirely exclude the occurrence of rare pathogenic CNVs and cn-LOH regions. Nevertheless, massively parallel sequencing of the whole genome from atherosclerotic lesions should be performed to provide more information regarding the involvement of somatic gene mutations in the development of the disease.

In conclusion, we discovered and confirmed amplification of the 10q24.31 (*ERLIN1*) and 12q24.11 (*UNG, ACACB*) genomic regions in these patients. It is possible that the rate of cell proliferation in arteries is lower than that of blood that continuously renews, providing more opportunities for genomic alterations over time in blood cells. Analysis of the DNA extracted from blood indicates a possible somatic origin for these CNVs. Taken together, the array CGH analysis of 10 patients with coronary artery disease and metabolic comorbidity using the Agilent SurePrint G3 Human CGH + SNP Microarray 2 × 400 K identified 90 different genomic structural rearrangements. Some CNVs and cn-LOH regions are enriched with genes previously associated with cardiovascular diseases and metabolic syndrome based on association studies.

## Methods

All experimental protocols were approved by the Research Institute of Medical Genetics Ethics Committee, and all methods were carried out in accordance with the approved guidelines. All study subjects provided written informed consent before participating. We analyzed with array-CGH ten Russian men (age 56.1 ± 3.0 years, mean ± SD) with severe 3-vessel coronary artery disease undergoing coronary artery bypass grafting. Each patient had a history of myocardial infarction prior to surgery and all components of metabolic syndrome (abdominal obesity, dyslipidaemia, hypertension, insulin resistance or type 2 diabetes mellitus). Thirty-three men (age 56.8 ± 7.3 years, mean ± SD) with coronary artery disease confirmed by angiography and undergoing coronary artery bypass grafting were included in the validation study (Table S4). All patients had metabolic syndrome defined according to the American Heart Association/National Heart Lung and Blood Institute modified National Cholesterol Education Program Adult Treatment Panel III guidelines, which require individuals to meet ≥3 of the following criteria: waist circumference >102 cm; triglyceride levels ≥1.7 mmol/L or treatment for dyslipidaemia; HDL levels <1.04 mmol/L or treatment for dyslipidaemia; blood pressure levels ≥130/85 mm Hg or treatment for hypertension; and fasting plasma glucose levels ≥6.1 mmol/L or treatment for type 2 diabetes mellitus^55^. Twenty-six patients (79%) had a history of myocardial infarction prior to surgery. Hypertension was diagnosed in 25 (76%) patients. Sixteen patients (48%) had hypercholesterolaemia, and nine patients had type 2 diabetes mellitus (27%).

Matched biopsy specimens were obtained from the CAP and IMA of thirty-three patients during coronary artery bypass graft surgery. Blood samples were obtained prior to the operation for all patients. The atherosclerotic lesions of the right coronary artery were classified as advanced according to the surgeon’s recommendations. Immediately after the operation, the samples were examined by a pathologist, cleaned of calcifications, fatty deposits and thrombotic material, and washed with sterile physiological saline solution. All samples were snap-frozen in liquid nitrogen and stored at −80 °C until further use.

Before the molecular cytogenetic analysis, several artery samples were stained with haematoxylin and eosin or immunostained with antibodies against smooth muscle-specific α-actin (clone 1A4, Dako, Glostrup, Denmark) and CD68 (clone KP1, Dako, Glostrup, Denmark). Smooth muscle cells were predominant in all analysed samples. Specimens collected from the CAP contained a large accumulation of macrophages to compare with the IMA.

DNA was then extracted from the arteries and blood using a QIAamp DNA Mini Kit (Qiagen, Valencia, CA, USA) as per the manufacturer’s instructions. In the present study, ten paired arteries (CAP and IMA) were analysed using array comparative genomic hybridization (array-CGH). Validation of selected CNVs in thirty-three paired arteries (CAP and IMA) and the blood of patients was performed using real-time PCR.

We performed the array-CGH using the Agilent SurePrint G3 Human CGH + SNP Microarray 2 × 400 K (G4842A, Agilent Technologies, Santa Clara, CA, USA). This microarray contains both 292 K CGH-probes and 120 K SNP-probes. DNA isolated from the arteries was hybridized against a sex-matched single reference DNA (Human Reference DNA-Male with Caucasian ethnicity, 5190-3796, Agilent Technologies, Santa Clara, CA, USA). After labelling with fluorescent dyes, the test and reference DNAs were hybridized on the microarray and washed and scanned according to manufacturer’s protocol.

The array-CGH data were normalized, centralized, and GC-corrected. All data were analysed using two programs in parallel to obtain high-confidence calls, Agilent Genomic Workbench v. 7.0 software (Agilent Technologies, Santa Clara, CA, USA) and DNAcopy package (R/Bioconductor). At least three consecutive probes were used to call a CNV. For the Agilent Genomic Workbench, the aberration algorithm of the Aberration Detection Method-2 was used with a minimum absolute average log2 ratio of 0.25 per region. Finally, CNVs with length <1 kb were also discarded. The other algorithm used to call CNVs was a circular binary segmentation algorithm implemented in the DNAcopy package of R/Bioconductor^56^. The default settings and a log2 ratio cut-off of −1.0 and 1.0 for loss and gain, respectively, was used to call CNVs. Any segment with an absolute median log2 ratio to median absolute deviation value less than 2 was filtered out. The CNVs detected with the Agilent Genomic Workbench and DNAcopy package for each individual were merged using outer probe boundaries. We defined a CNV as ‘high-confidence’ if it was detected by both algorithms at the sample level. We also visually inspected each chromosome for DNA aberrations.

CNV calls were compared to the general population variants using the Database of Genomic Variants, build 37 (hg19) (DGV), and the array-CGH database at the Laboratory of Cytogenetics (Institute of Medical Genetics), which comprises 123 analysed patient samples and publications indexed in PubMed. CNV calls were considered novel if they did not overlap with more than 50% of the genome locus of previously reported CNV regions in the DGV. Functional enrichment analysis of genes located in CNVs and cn-LOH was performed using the web-based GEne SeT AnaLysis Toolkit^57^. The reference gene set comprised the human genome genes. Kyoto Encyclopedia of Genes and Genomes (KEGG) categories were determined using a hypergeometric statistical test. P-values were corrected for multiple testing using FDR. We also used the HuGE Navigator Database to identify disease-gene association with the cardiovascular diseases.

CNVs of interest were validated using TaqMan copy number probes Hs00986678_cn and Hs06959694_cn (Life Technologies, California, USA), according to the manufacturer’s standard protocol. RNaseP locus was used as internal control for gDNA copy-number standardization (TaqMan Copy Number Reference Assay RNase P, reference 4403328). Genomic real-time quantitative PCR analysis (qPCR) was performed using the AriaMx Real-Time PCR System (Agilent Technologies, Santa Clara, CA, USA). The Human Reference DNA-Male with Caucasian ethnicity (5190-3796, Agilent Technologies, Santa Clara, CA, USA) sample was used as a calibrator. All samples within each run were assayed in triplicate and averaged to determine the copy number. For each assay, a standard curve was run and recorded. A relative quantification analysis was performed using the Pfaffl method considering the amplification efficiencies of each primer pair^58^. Values in the range of 0.8–1.2 indicated the presence of two copies, <0.6 indicated a copy number loss and >1.4 was considered a gain.

## Additional Information

**How to cite this article**: Nazarenko, M. S. *et al*. Genomic structural variations for cardiovascular and metabolic comorbidity. *Sci. Rep.* **7**, 41268; doi: 10.1038/srep41268 (2017).

**Publisher's note:** Springer Nature remains neutral with regard to jurisdictional claims in published maps and institutional affiliations.

## Supplementary Material

Supplementary Material

## Figures and Tables

**Table 1:** DNA copy number changes in arteries of patients with coronary artery disease and metabolic comorbidity.

№	Patient ID	Tissue	ISCN, 2013	Length (bp)	Contained genes	DGV^1^	Affected patients in the array-CGH database^2^	Previous cardiovascular and metabolic disease associations for gene	PMID
1	1, 2, 5, 7, 8, 9, 10	CAP, IMA	arr[hg19]1p22.2 (89474710–89479067)×3	4358	***GBP3***	Yes	0	Carotid artery intima media thickness over 10 years	26343869
2	2, 3, 4, 6, 7, 8, 10	CAP, IMA	arr[hg19]1p21.1 (104115870–104120646)×1	4777	***AMY2B***	Yes	6	Type 2 diabetes and T2D-associated end-stage renal disease	24707315
3	2, 3, 4, 7	CAP, IMA	arr[hg19]1q31.3 (196748499–196800758)×1	52260	***CFHR3**,**CDHR1***	Yes	0	Blood pressure and hypertension; IgA nephropathy	22848687 25205734
4	4	CAP, IMA	arr[hg19]2q13 (110849200–110980342)×1	131143	*MALL, **NPHP1**, NCRNA00116*	Yes	1	Juvenile nephronophthisis; Joubert syndrome type 4; Senior-Loken syndrome 1; Bardet-Biedl syndrome	24746959
5	4	CAP, IMA	arr[hg19]2q37.3 (242865920–243076752)×1	210833	***LOC105373979** LOC728323*	Yes	0	Coronary artery calcification	23870195
6	1, 7	CAP, IMA	arr[hg19]3p21.1 (53028096–53059285)×1	31190	***SFMBT1***	Yes	0	Hypertension	22479346
7	1	CAP, IMA	arr[hg19]7q33 (133764157–133812235)×1	48079	***EXOC4**, LRGUK*	Yes	0	Type 2 Diabetes and fasting glucose, obesity, metabolic syndrome	18498660
8	7	CAP, IMA	arr[hg19] 9p21.1 (28591992–28758126)×1	166135	***LINGO2***	Yes	0	Body mass	25711307
9	2	CAP, IMA	arr[hg19]10q21.1 (56457621–56465378)×3	7758	***PCDH15***	Yes	0	Familial combined hyperlipidemia; carotid artery intima media thickness over 10 years	19816713 26343869
10	1	CAP, IMA	arr[hg19]10q21.3 (68078402–68109984)×1	31583	***CTNNA3***	Yes	2	Arrhythmogenic right ventricular cardiomyopathy; Cervical artery dissection	2313640 22617347
11	10	CAP, IMA	arr[hg19]10q24.31 (101911972–101944947)×3	32976	***ERLIN1***	No	0	Nonalcoholic fatty liver disease; plasma levels of alanine-aminotransferase; hemorrhagic stroke	23477746 18940312 20198315
12	4	CAP, IMA	arr[hg19]12q24.11 (109547804–109661604)×3	113801	*UNG, **ACACB***	Yes	0	Type 2 diabetes-associated nephropathy; body mass index; obesity; type 2 diabetes in postmenopausal women; C-reactive protein level in a prediabetic and diabetic population; metabolic syndrome	23460794 21908218 21553357 20855566 26030797
13	4	CAP, IMA	arr[hg19]16q13.11–q13.12 (15048751–15122499)×1	73749	***PDXDC1***	Yes	0	Metabolic syndrome	24981077
14	4, 10	CAP, IMA	arr[hg19]16q22.1 (70152776–70190625)×3	37850	***PDPR***	Yes	3	Coronary artery disease	20808825
15	10	CAP, IMA	arr[hg19]17p11.2 (21736281–22154574)×3	418294	***MTRNR2L1**, FAM27L, FLJ36000*	Yes	0	Insulin-resistant obese subjects	26376914
16	3	CAP, IMA	arr[hg19]22q11.23 (24347959–24376216)×1	28258	***GSTT1**, LOC391322*	Yes	5	Hypertension	24788870 27686690 20517701

^1^Overlapping status with DGV (http://projects.tcag.ca/variation/) entries.

^2^Affected patients according to the array-CGH database at the Laboratory of Cytogenetics, Institute of Medical Genetics. Candidate genes for cardiovascular diseases and their risk factors are marked in Table 1 by bold type. Abbreviations: CAP - right coronary artery in the area of advanced atherosclerotic plaque; IMA - intact internal mammary artery; ISCN - International System for Human Cytogenetic Nomenclature.

**Table 2:** DNA copy-neutral changes in arteries of patients with coronary artery disease and metabolic comorbidity.

№	Patient ID	Tissue	ISCN, 2013	Length (bp)	Contained genes
1	1	CAP, IMA	arr[hg19]7q21.12–q21.13 (86765642–89595238)×2 hmz	2829597	*DMTF1, TMEM243, TP53TG1, CROT, ABCB4, **ABCB1**, RUNDC3B, DBF4, ADAM22, SRI, STEAP4, ZNF804B, C7orf62*
			arr[hg19]11p12 (37110535–40336165)×2 hmz	3225631	*LRRC4C*
			arr[hg19]18q12.1–q12.2 (29841532–33526175)×2 hmz	3684644	*GAREM, WBP11P1, KLHL14, CCDC178, ASXL3, NOL4, DTNA, MAPRE2, ZNF397, ZSCAN30, ZNF271, ZNF24, ZNF396, INO80C, MIR3975, GALNT1, MIR187*
2	2	CAP, IMA	arr[hg19]6q15 (90703280–92347529)×2 hmz	1644250	*BACH2, MIR4464, MAP3K7, MIR4643, CASC6*
			arr[hg19]8p11.1–p11.21 (40893397–43520356)×2 hmz	2627000	*SFRP1, GOLGA7, GINS4, NKX6-3, ANK1, MIR486, KAT6A, AP3M2, **PLAT**, IKBKB, VDAC3, SLC20A2, POLB, DKK4, C8orf40, CHRNB3, CHRNA6, MIR4469, THAP1, RNF170, HOOK3, FNTA, SGK196, POTEA, HGSNAT*
			arr[hg19]8q11.1–q11.21 (46940022–49990006)×2 hmz	3049985	*LINC00293, LOC100287846, KIAA0146, CEBPD, PRKDC, MCM4, UBE2V2, EFCAB1, SNAI2, C8orf22*
3	3	CAP, IMA	arr[hg19]17q23.1–q24.1 (57734690–64120329)×2 hmz	6385640	*CLTC, PTRH2, TMEM49, MIR21, TUBD1, RPS6KB1, RNFT1, DHX40P, HEATR6, LOC645638, LOC653653, CA4, USP32, SCARNA20, C17orf64, APPBP2, PPM1D, BCAS3, TBX2, C17orf82, TBX4, NACA2, BRIP1, INTS2, MED13, TBC1D3P2, EFCAB3, METTL2A, TLK2, MRC2, MARCH10, TANC2, CYB561, **ACE**, KCNH6, DCAF7, TACO1, MAP3K3, LIMD2, STRADA, CCDC47, DDX42, FTSJ3, PSMC5, SMARCD2, TCAM1, CSH2, GH2, CSH1, CSHL1, **GH1**, CD79B, SCN4A, C17orf72, ICAM2, ERN1, SNORD104, SNORA76, TEX2, **PECAM1**, C17orf60, POLG2, DDX5, CCDC45, SMURF2, LOC146880, PLEKHM1P, LRRC37A3, FLJ32065, GNA13, **RGS9**, AXIN2, CCDC46*
4	7	CAP, IMA	arr[hg19]9p21.2 (26038217–27819206)×2 hmz	1780990	*LOC100506422, CAAP1, PLAA, IFT74, LRRC19, TEK, LINC00032, EQTN, MOB3B, IFNK, C9orf72*

Candidate genes for cardiovascular diseases according to the HuGE Navigator Database (https://phgkb.cdc.gov/HuGENavigator/home.do) are marked in Table 2 by bold type. Abbreviations: CAP - right coronary artery in the area of advanced atherosclerotic plaque, IMA - intact internal mammary artery; ISCN - International System for Human Cytogenetic Nomenclature.
