# Supplementary material for: Genomic structural variations for cardiovascular and metabolic comorbidity
Source: Sci Rep. 2017 Jan 25;7:41268. doi: 10.1038/srep41268 (PMC5264603; doi:10.1038/srep41268)
Supplement: Supplementary Material [file srep41268-s1.doc]

**Correspondence and requests for materials:**

maria.nazarenko@medgenetics.ru

**Title:**

Genomic structural variations for cardiovascular and metabolic comorbidity

Maria S. Nazarenko*^1,3^, Aleksei A. Sleptcov^1,3^, Igor N. Lebedev^2,3^, Nikolay A. Skryabin^2,3^, Anton V. Markov^1,3^, Maria V. Golubenko^1,3,5^, Iuliya A. Koroleva^1^, Anton N. Kazancev^4^, Olga L. Barbarash^4^, Valery P. Puzyrev^1,3^

^1^Laboratory of Population Genetics, Research Institute of Medical Genetics, Tomsk National Research Medical Center, Russian Academy of Sciences, Tomsk, Russia;

^2^Laboratory of Cytogenetics, Research Institute of Medical Genetics, Tomsk National Research Medical Center, Russian Academy of Sciences, Tomsk, Russia;

^3^Laboratory of Human Ontogenetics, Tomsk State University, Tomsk, Russia;

^4^Department of Multifocal Atherosclerosis, Research Institute for Complex Issues of Cardiovascular Diseases, Kemerovo, Russia;

^5^Department of Experimental and Clinical Cardiology, Research Institute for Complex Issues of Cardiovascular Diseases, Kemerovo, Russia.

**SUPPLEMENTAL MATERIAL**

**Contents**

**Supplemental Tables**

| Table S1. Gains and losses detected for each of the ten patients using the Agilent array-CGH | 2 |
| --- | --- |
| Table S2. WebGestalt-generated enrichment of KEGG categories for genic CNVs | 10 |
| Table S3. WebGestalt-generated enrichment of KEGG categories for genic cn-LOH | 11 |
| Table S4. Baseline characteristics of the study subjects | 12 |

# Supplementary Table S1. Gains and losses detected for each of the ten patients using the Agilent array-CGH. For each gain or loss the information about its genomic coordinates (alignment to the human reference genome 19 (build GRCh37: Feb. 2009, hg19)), length, average log2 ratio, and contained genes is indicate.

| **Num** | **Individual** | **Chr** | **Length** | **Start** | **Stop** | **log2ratio** | **Total** | **Genes** |
| --- | --- | --- | --- | --- | --- | --- | --- | --- |
| 1 | 10 | 1p36.21 | 19598 | 15788935 | 15808532 | -1.01 | 1 | *CELA2A, CELA2B* |
| 2 | 1, 2, 3, 4, 6, 7, 9, 10 | 1p36.11 | 63975 | 25592988 | 25656962 | -1.35, -0.71, -0.63, -0.76, -0.58, -0.76, -0.61, -1.33 | 8 | *RHD* |
| 3 | 1, 2, 5, 7, 8, 9, 10 | 1p22.2 | 4358 | 89474710 | 89479067 | 0.69, 0.72, 0.54, 0.73, 0.55, 1.01, 0.59 | 7 | *GBP3* |
| 4 | 2, 3, 4, 6, 7, 8, 10 | 1p21.1 | 4777 | 104115870 | 104120646 | -0.47, -0.7, -0.38, -0.58, -0.44, -0.75, -0.42 | 7 | *AMY2B* |
| 5 | 1, 2, 3, 4, 6, 7, 8, 10 | 1p13.1 | 22909 | 117175226 | 117198134 | -0.55, -0.72, -0.66, -0.91, -0.63, -0.72, -0.38, -0.73 | 8 | *IGSF3* |
| 6 | 2, 3, 7, 9 | 1q21.2 | 609721 | 149041933 | 149651653 | -0.42, -0.51, -0.23, -0.31 | 4 | *LOC101929780, NBPF23, LOC388692, LINC00869, LINC00623, FCGR1C, PPIAL4B, PPIAL4A, PPIAL4C* |
| 7 | 3 | 1q21.3 | 13552 | 152573149 | 152586700 | -4.15 | 1 | *LCE3C, LCE3B* |
| 8 | 1, 5, 6, 8, 9, 10 | 1q31.3 | 52260 | 196748499 | 196800758 | 0.47, 0.59, 0.76, 0.86, 0.97, 0.72 | 6 | *CFHR3, CFHR1* |
| 9 | 1, 7 | 1q44 | 50643 | 248738898 | 248789540 | -0.84, -2.58 | 2 | *OR2T10, OR2T11* |
| 10 | 1, 2, 3, 4, 5, 6, 7, 8, 9, 10 | 2p11.2 | 576814 | 87364070 | 87940883 | -0.4, -0.49, -0.4, -0.26, -0.65, -0.8,  -0.48, -0.36, -1.05, -0.56 | 10 | *LINC00152, MIR4435-1* |
| 11 | 1, 2, 3, 4, 5, 6, 7, 8, 9, 10 | 2p11.2 | 156058 | 89163862 | 89319919 | 0.78, 0.49, 0.79, 0.69, 0.67, 0.81, 0.83, 0.69, 0.9, 0.83 | 10 |  |
| 12 | 4 | 2q13 | 131143 | 110849200 | 110980342 | -1.09 | 1 | *LINC00116, MALL, NPHP1* |
| 13 | 9 | 2q14.1 | 126038 | 117007542 | 117133579 | -1.33 | 1 |  |
| 14 | 1, 3, 4, 6, 7, 10 | 2q31.2 | 11194 | 179296800 | 179307993 | 0.5, 0.49, 0.52, 0.55, 0.9, 0.52 | 6 | *PRKRA* |
| 15 | 4 | 2q37.3 | 210833 | 242865920 | 243076752 | -1.05 | 1 | *LOC728323* |
| 16 | 9 | 3p21.31 | 27268 | 46821461 | 46848728 | -1.3 | 1 |  |
| 17 | 1, 7 | 3p21.1 | 31190 | 53028096 | 53059285 | -0.94, -0.87 | 2 | *SFMBT1* |
| 18 | 10 | 3q12.2 | 105434 | 100342240 | 100447673 | 0.62 | 1 | *GPR128, TFG* |
| 19 | 3 | 3q25.1 | 32018 | 151513431 | 151545448 | -1.21 | 1 | *MIR548H2; AADAC* |
| 20 | 1, 2, 3, 4, 5, 6, 7, 8, 9, 10 | 3q26.1 | 104608 | 162514534 | 162619141 | 3.44, 4.16, 3.01, 4.75, 3.84, 3.81, 4.5, 3.8, 4.12, 4.62 | 10 |  |
| 21 | 1, 2, 3, 7, 8 | 3q29 | 38550 | 195425875 | 195464424 | -0.35, -0.36, -0.67, -1.42, -0.67 | 5 | *MIR570, MUC20* |
| 22 | 1, 4, 5, 6, 7, 9, 10 | 4p15.1 | 112290 | 34700703 | 34812992 | 0.81, 1.1, 1.1, 0.74, 1.16, 1.15, 0.92 | 7 |  |
| 23 | 2, 3, 4, 5, 6, 8 | 4q13.2 | 116106 | 69374729 | 69490834 | -0.9, -0.87, -2.96, -1.03, -0.88, -0.9 | 6 | *UGT2B17* |
| 24 | 5 | 4q13.2 | 107146 | 70152637 | 70259782 | -1.4 | 1 | *UGT2B28* |
| 25 | 6 | 5p14.3 | 82012 | 21286941 | 21368952 | -0.65 | 1 |  |
| 26 | 6, 7 | 5q13.2 | 1369271 | 69288477 | 70657747 | -0.69, -0.29 | 2 | *SERF1B, SMN2, SERF1A, SMN1, NAIP, GTF2H2, GTF2H2B, SMA4, SMA5, LOC441081, GUSBP9* |
| 27 | 6 | 5q23.1 | 56746 | 120102313 | 120159058 | -0.85 | 1 |  |
| 28 | 2, 3, 6, 8, 9 | 5q35.3 | 13736 | 180416012 | 180429747 | -0.68, -0.65, -0.76, -1.42, -0.73 | 5 | *BTNL3* |
| 29 | 1, 2, 6, 8, 10 | 6p22.2 | 108842 | 26830188 | 26939029 | -0.58, -0.53, -0.98, -0.98, -0.85 | 5 | *GUSBP2, LINC00240* |
| 30 | 2, 10 | 6p21.33 | 32288 | 31277799 | 31310086 | -1.3, -1.01 | 2 |  |
| 31 | 1, 3, 4, 5, 6, 7, 8, 9, 10 | 6p21.32 | 8607 | 32460308 | 32468914 | 1.41, 1.1, 1.05, -1.14, 0.94, 1.14, 1, -1.07, 1.11 | 9 |  |
| 32 | 1, 2, 3, 4, 5, 6, 7, 8, 9, 10 | 6p21.32 | 7667 | 32480027 | 32487693 | -1.03, -0.94, -0.98, -1.34, -0.58, -0.99, -1.02, -1.11, -0.94, -0.98 | 10 | *HLA-DRB5* |
| 33 | 5, 9 | 6p21.32 | 24608 | 32497322 | 32521929 | -1.26, -0.81 | 2 | *HLA-DRB5, HLA-DRB6* |
| 34 | 1, 2, 3, 4, 5, 6, 7, 8, 9, 10 | 6p11.2 | 115103 | 57360339 | 57475441 | 0.69, 1.08, 1.19, 0.97, 0.99, 1.06, 0.83, 1.15, 0.71, 1.31 | 10 | *PRIM2* |
| 35 | 1, 2, 3, 5, 7, 8 | 6q14.1 | 66088 | 78979172 | 79045259 | -3, -3, -1, -0.91, -0.52, -0.91 | 6 |  |
| 36 | 6 | 6q21 | 71776 | 110341761 | 110413536 | -0.56 | 1 |  |
| 37 | 1, 2, 3, 4, 5, 6, 7, 8, 9, 10 | 7p15.2 | 36332 | 26893878 | 26930209 | 0.68, 0.7, 0.89, 0.83, 0.75, 0.91, 0.87, 0.84, 0.8, 0.84 | 10 | *SKAP2* |
| 38 | 1, 2, 3, 4, 5, 6, 7, 8, 9, 10 | 7p14.1 | 27536 | 41280084 | 41307619 | 1.15, 0.69, 0.89, 1.07, 1.04, 1.04, 1.09, 0.96, 1.03, 1.11 | 10 |  |
| 39 | 9 | 7p12.1 | 122103 | 53461023 | 53583125 | -1.17 | 1 |  |
| 40 | 8 | 7p12.1 | 226776 | 53684365 | 53911140 | -1.03 | 1 | *LINC01446* |
| 41 | 2, 3, 4, 5, 6, 7, 8, 9, 10 | 7q33 | 48079 | 133764157 | 133812235 | 1.11, 0.88, 0.92, 0.74, 1.18, 1.28, 0.93, 1.14, 0.94 | 9 | *LRGUK* |
| 42 | 1, 2, 3, 4, 5, 6, 7, 8, 9, 10 | 8p23.1 | 840430 | 7239491 | 8079920 | -0.64, -0.75, -0.67, -0.49, -0.67, -0.79, -1.19, -0.58, -0.77, -0.85 | 10 | *DEFB4B, DEFB103B, DEFB103A, SPAG11B, DEFB104B, DEFB104A, DEFB106A, DEFB106B, DEFB105B, DEFB105A, DEFB107B, DEFB107A, PRR23D1, PRR23D2, SPAG11A, DEFB103A, DEFB103B, DEFB4A, ZNF705B, FAM90A10P, PRR23D2, PRR23D1, FAM66E, USP17L8, USP17L3, DEFB109P1B, MIR548I3* |
| 43 | 1, 2, 3, 4, 5, 6, 7, 8, 9, 10 | 8p22 | 7217 | 15403439 | 15410655 | 1.23, 0.89, 0.85, 1.2, 1.37, 0.91, 1.09, 1.16, 0.79, 0.96 | 10 | *TUSC3* |
| 44 | 2, 5, 6, 7, 9, 10 | 8p11.22 | 108042 | 39237438 | 39345479 | -1.01, -0.93, -1, -1.1, -2.49, -0.95 | 6 | *ADAM5, ADAM3A* |
| 45 | 2, 4, 5, 10 | 8q24.3 | 10852 | 144703546 | 144714397 | -1.28, -1.28, -1.08, -0.88 | 4 |  |
| 46 | 1, 2, 3, 4, 5, 6, 7, 8, 9, 10 | 9p24.1 | 30730 | 5304850 | 5335579 | -0.71, -0.68, -0.67, -0.55, -0.71, -0.74, -0.82, -0.7, -0.9, -0.75 | 10 | *RLN1* |
| 47 | 7 | 9p21.1 | 166135 | 28591992 | 28758126 | -0.94 | 1 | *LINGO2* |
| 48 | 4 | 9p13.1-p12 | 295602 | 41979244 | 42274845 | 0.64 | 1 | *KGFLP2, LINC01189* |
| 49 | 2, 3, 4, 6, 7 | 9p12-p11.2 | 330527 | 43505843 | 43836369 | -0.67, -0.65, -0.53, -0.67, -1 | 5 | *SPATA31A6, CNTNAP3B* |
| 50 | 6 | 10q11.21 | 111348 | 45247685 | 45359032 | 0.69 | 1 | *TMEM72-AS1* |
| 51 | 4, 5, 6, 7 | 10q11.22 | 1343460 | 46359751 | 47703210 | -0.32, -0.33, -0.4, -0.42 | 4 | *PTPN20A, PTPN20B, GLUD1P7, BMS1P5, BMS1P1, SYT15, FAM35BP, GPRIN2, NPY4R, LOC100996758 LINC00842, ANXA8, FAM25C, AGAP9, BMS1P6, BMS1P2, FAM25G, HNRNPA1P33, FAM350P, ANTXRLP1, ANTXRL* |
| 52 | 2 | 10q21.1 | 7758 | 56457621 | 56465378 | 1.17 | 1 | *PCDH15* |
| 53 | 1 | 10q21.3 | 31583 | 68078402 | 68109984 | -1.06 | 1 | *CTNNA3* |
| 54 | 10 | 10q24.31 | 32976 | 101911972 | 101944947 | 0.83 | 1 | *ERLIN1* |
| 55 | 1, 2, 3, 4, 5, 6, 7, 8, 9, 10 | 11p15.1 | 8100 | 18955543 | 18963642 | 0.71, 1.02, 1.03, 1.31, 1.02, 1.16, 0.88, 0.99, 1, 1.21 | 10 | *MRGPRX1* |
| 56 | 1, 3, 5 | 11q11 | 34694 | 55371188 | 55405881 | -0.95, -0.85, -1.07 | 3 | *OR4C11, OR4P4* |
| 57 | 1, 3, 4, 5, 6, 7, 8, 9, 10 | 12p13.31 | 76103 | 9637323 | 9713425 | -1,  -0.98, -4.28, -1, -0.61, -1, -0.87, -0.74, -0.93 | 9 |  |
| 58 | 1, 2, 3, 5, 6, 7, 8, 9, 10 | 12p13.2 | 39698 | 11507315 | 11547012 | 0.5, 0.39, 0.64, 0.47, 0.57, 0.78, 0.66, 0.77, 1.01 | 9 | *PRB1, PRB2* |
| 59 | 6 | 12q21.31 | 30267 | 83177976 | 83208242 | -0.92 | 1 | *TMTC2* |
| 60 | 4 | 12q24.11 | 113801 | 109547804 | 109661604 | 0.53 | 1 | *UNG, ACACB* |
| 61 | 3 | 13q12.11 | 22968 | 20774181 | 20797148 | 0.65 | 1 | *GJB6* |
| 62 | 6 | 14q11.2 | 1044916 | 19376762 | 20421677 | 0.71 | 1 | *OR11H12, POTEG, POTEM, OR4N2, OR4Q3, OR4M1, OR4K2, OR4K5, OR4K1, BMS1P17, BMS1P18, OR11H2, LOC642426* |
| 63 | 2, 3, 4, 7, 8, 10 | 14q11.2 | 859537 | 19562141 | 20421677 | -0.52, -0.56, -0.36, -0.39, -0.2, -0.52 | 6 | *POTEG, POTEM, OR4N2, OR4Q3, OR4M1, OR4K2, OR4K5, OR4K1, BMS1P17, BMS1P18, OR11H2* |
| 64 | 7 | 14q21.1 | 463748 | 41554068 | 42017815 | -0.94 | 1 | *LOC644919* |
| 65 | 1, 2, 3, 5, 7, 10 | 14q24.3 | 20674 | 74001651 | 74022324 | 1.1, 1, 0.94, 0.87, 1.01, 0.9 | 6 | *HEATR4, ACOT1* |
| 66 | 2 | 14q32.33 | 223397 | 106027750 | 106251146 | 0.54 | 1 | *ELK2AP, MIR8071-1, MIR8071-2* |
| 67 | 1, 2, 3, 4, 5, 6, 7, 8, 9, 10 | 14q32.33 | 879987 | 106334907 | 107214893 | 1.13, 1, 0.88, 2.44, 0.59, 1.44, 1.45, 0.88, 1.92, 1.93 | 10 | *ADAM6, KIAA0125, LINC00226, LINC00221* |
| 68 | 2, 3, 4, 5, 8, 9 | 15q11.1-q11.2 | 2077055 | 20481702 | 22558756 | -0.48, -0.6, -0.41, -0.44, 0.79, -0.86 | 6 | *GOLGA6L6, POTEB2, POTEB, OR4M2, OR4N4, CHEK2P2, HERC2P3, NBEAP1, NF1P2, CT60, CXADRP2, LOC646214, LOC727924, REREP3* |
| 69 | 6 | 15q26.3 | 21279 | 102161480 | 102182758 | -0.88 | 1 | *TM2D3* |
| 70 | 4 | 16q13.12-q13.11 | 73749 | 15048751 | 15122499 | -0.98 | 1 | *PDXDC1* |
| 71 | 2, 3, 5, 8, 9 | 16p11.2 | 1318564 | 31986127 | 33304690 | -0.52, -0.52, -0.41, -0.11, -1 | 5 | *HERC2P4, SLC6A10P, TP53TG3D, TP53TG3, TP53TG3C, TP53TG3B, LOC390705* |
| 72 | 7, 10 | 16p11.2-16p11.1 | 299493 | 34427800 | 34727292 | 0.73, 0.58 | 2 | *LOC283914, LOC146481* |
| 73 | 4, 10 | 16q22.1 | 37850 | 70152776 | 70190625 | 1.13, 1.01 | 2 | *PDPR* |
| 74 | 1, 2, 7 | 16q23.1 | 100303 | 74372285 | 74472587 | -0.52, -0.81, -0.58 | 3 | *LOC283922, CLEC18B* |
| 75 | 4, 10 | 16q23.2 | 35057 | 74372285 | 74407341 | 0.76, 0.83 | 2 | *LOC283922* |
| 76 | 10 | 17p11.2 | 418294 | 21736281 | 22154574 | 0.65 | 1 | *MTRNR2L1, FAM27L, FLJ36000* |
| 77 | 2, 4, 6, 8, 10 | 17q21.31 | 47930 | 44221743 | 44269672 | -0.72, -0.56, 0.55, 0.6, -0.66 | 5 | *KANSL1* |
| 78 | 2 | 17q22 | 286757 | 50983095 | 51269851 | -1.2 | 1 | *C17orf112* |
| 79 | 4, 5, 6, 7, 9, 10 | 20p13 | 25136 | 1559350 | 1584485 | 1.13, 2.13, 3.14, 2, 1.33, 4 | 6 | *SIRPB1* |
| 80 | 2, 4, 6, 8, 9 | 21p11.2-21p11.1 | 240478 | 10701593 | 10942070 | -0.4, -0.44, -0.34, -0.37, -0.29 | 5 | *TPTE* |
| 81 | 4, 6, 7 | 22q11.1 | 235187 | 16111396 | 16346582 | -1, 0.4, -0.7 | 3 | *POTEH, BMS1P18, BMS1P17* |
| 82 | 1, 2, 3, 4, 6, 7, 8, 10 | 22q11.21 | 147639 | 18729944 | 18877582 | -0.85, -0.99, -0.68, -1.04, -0.61, -0.86, -0.63, -0.81 | 8 | *GGT3P* |
| 83 | 1, 4, 5, 6 | 22q11.23 | 42296 | 24347959 | 24390254 | 0.98, 1.02, 0.95, 1.04 | 4 | *GSTT1, GSTTP2, LOC391322* |
| 84 | 3 | 22q11.23 | 28258 | 24347959 | 24376216 | -1.01 | 1 | *GSTT1, LOC391322* |
| 85 | 7 | 22q13.1 | 20469 | 39365037 | 39385505 | -0.78 | 1 | *APOBEC3B, APOBEC3A* |
| 86 | 6 | Xp11.4 | 41575 | 38374142 | 38415716 | -0.76 | 1 |  |
| 87 | 1, 2, 4, 6 | Xp11.1 | 91093 | 58206905 | 58297997 | 0.94, 0.9, 1.34, 0.78 | 4 |  |
| 88 | 2 | Xq22.2 | 43521 | 103261576 | 103305096 | 0.91 | 1 | *H2BFWT, H2BFM, MIR1256* |
| 89 | 4 | Xq26.3 | 42357 | 134757970 | 134800326 | 1.16 | 1 |  |
| 90 | 5, 6, 8 | Yq11.233-q11.23 | 1543815 | 24891284 | 26435098 | -0.85, -0.66, -0.62 | 3 | *CDY1B, CDY1, TTTY17A, TTTY17B, TTTY17C, TTTY4, TTTY4B, TTTY4C, TTTY3, TTTY3B, BPY2, BPY2C, BPY2B, DAZ1, DAZ2, DAZ3, DAZ4, GOLGA2P2Y, GOLGA2P3Y, CSPG4P1Y* |

# Supplementary Table S2. WebGestalt-generated enrichment of KEGG categories for genic CNVs.

The table provide the number of reference genes in the category (C), number of genes in the gene set and also in the category (O), expected number in the category (E), Ratio of enrichment (R), p value from hypergeometric test (rawP), and p value adjusted by multiple test adjustment.

| **PathwayName** | **Num genes** | **Gene Symbol** | **Statistics** |
| --- | --- | --- | --- |
| Olfactory transduction | 12 | *OR2T10, OR4P4, OR4K2, OR2T11, OR4N2, OR4C11, OR4K1, OR4M1, OR4Q3, OR4K5, OR4M2, OR4N4* | C=388;O=12;E=1.71;R=7.02; rawP=1.83e-07;adjP=3.29e-06 |
| Starch and sucrose metabolism | 3 | *AMY2B, UGT2B17, UGT2B28* | C=54;O=3;E=0.24;R=12.61; rawP=0.0018;adjP=0.0162 |
| Metabolism of xenobiotics by cytochrome P450 | 3 | *GSTT1, UGT2B17, UGT2B28* | C=71;O=3;E=0.31;R=9.59; rawP=0.0039;adjP=0.0189 |
| Drug metabolism - cytochrome P450 | 3 | *GSTT1, UGT2B17, UGT2B28* | C=73;O=3;E=0.32;R=9.33; rawP=0.0042;adjP=0.0189 |
| Ascorbate and aldarate metabolism | 2 | *UGT2B17, UGT2B28* | C=26;O=2;E=0.11;R=17.46; rawP=0.0059;adjP=0.0212 |
| Pancreatic secretion | 3 | *CELA2A, AMY2B, CELA2B* | C=101;O=3;E=0.44;R=6.74; rawP=0.0102;adjP=0.0262 |
| Pentose and glucuronate interconversions | 2 | *UGT2B17, UGT2B28* | C=32;O=2;E=0.14;R=14.19; rawP=0.0088;adjP=0.0262 |
| Porphyrin and chlorophyll metabolism | 2 | *UGT2B17, UGT2B28* | C=43;O=2;E=0.19;R=10.56; rawP=0.0155;adjP=0.0349 |
| Other types of O-glycan biosynthesis | 2 | *UGT2B17, UGT2B28* | C=46;O=2;E=0.20;R=9.87; rawP=0.0176;adjP=0.0352 |
| Systemic lupus erythematosus | 3 | *H2BFWT, HLA-DRB5, H2BFM* | C=136;O=3;E=0.60;R=5.01;rawP=0.0225;adjP=0.0368 |
| Drug metabolism - other enzymes | 2 | *UGT2B17, UGT2B28* | C=52;O=2;E=0.23;R=8.73; rawP=0.0222;adjP=0.0368 |
| Steroid hormone biosynthesis | 2 | *UGT2B17, UGT2B28* | C=56;O=2;E=0.25;R=8.11; rawP=0.0254;adjP=0.0381 |
| Retinol metabolism | 2 | *UGT2B17, UGT2B28* | C=64;O=2;E=0.28;R=7.09; rawP=0.0326;adjP=0.0451 |

# Supplementary Table S3. WebGestalt-generated enrichment of KEGG categories for genic cn-LOH..

The table provide the number of reference genes in the category (C), number of genes in the gene set and also in the category (O), expected number in the category (E), Ratio of enrichment (R), p value from hypergeometric test (rawP), and p value adjusted by multiple test adjustment.

| **PathwayName** | **Num genes** | **Gene Symbol** | **Statistics** |
| --- | --- | --- | --- |
| RIG-I-like receptor signaling pathway | 3 | *MAP3K7, IKBKB, IFNK* | C=71;O=3;E=0.24;R=12.74; rawP=0.0017;adjP=0.0165 |
| Neuroactive ligand-receptor interaction | 5 | *CHRNA6, GH2, CSH1, CHRNB3, GH1* | C=272;O=5;E=0.90;R=5.54; rawP=0.0022;adjP=0.0165 |
| Jak-STAT signaling pathway | 4 | *GH2, CSH1, GH1, IFNK* | C=155;O=4;E=0.51;R=7.78; rawP=0.0018;adjP=0.0165 |
| Wnt signaling pathway | 4 | *SFRP1, MAP3K7, AXIN2, DKK4* | C=150;O=4;E=0.50;R=8.04; rawP=0.0016;adjP=0.0165 |
| Cell cycle | 3 | *MCM4, PRKDC, DBF4* | C=124;O=3;E=0.41;R=7.30; rawP=0.0083;adjP=0.0470 |
| ABC transporters | 2 | *ABCB4, ABCB1* | C=44;O=2;E=0.15;R=13.71; rawP=0.0094;adjP=0.0470 |

**Table S4.** Baseline characteristics of the study subjects.

| Characteristic | All patients, n=33 |
| --- | --- |
| Mean age±SD, years | 56.8±7.3 |
| BMI±SD, kg/m^2^ | 28.9±3.8 |
| Miocardial infarction, n (%) | 26 (79) |
| Hypertension, n (%) | 25 (76) |
| Hypercholesterolemia, n (%) | 16 (48) |
| Smoking, n (%) | 16 (48) |
| Diabetes mellitus, n (%) | 9 (27) |
| Glucose (mmol/L) | 6.2±1.5 |
| Total cholesterol (mmol/L) | 5.3±1.2 |
| Triglycerides (mmol/L) | 2.6±2.0 |
